# Infertility costs and associated factors in childhood cancer survivors: the role of radiotherapy

**DOI:** 10.1007/s11764-026-02032-2

**Published:** 2026-05-08

**Authors:** Bastien Bourrion, Henri Panjo, Brice Fresneau, Laura Lenez, Florent de Vathaire, Mathilde François, Nathalie Pelletier-Fleury

**Affiliations:** 1 INSERM, Université Paris-Saclay, UVSQ, CESP, Hôpital Paul Brousse, bâtiment 15/16 Inserm, 16 Avenue Paul Vaillant Couturier, 94807 Villejuif Cedex, France; 2 Département de Médecine Générale, Faculté des Sciences de la Santé Simone Veil, UVSQ, 2 Avenue de la Source de la Bièvre, 78180 Montigny le Bretonneux, France; 3 Radiation Epidemiology Group, University of Paris Saclay, Gustave Roussy, Inserm, Unit 1018-CESP, Villejuif, France; 4 Département de Cancérologie de l’Enfant et de l’Adolescent, Gustave Roussy, F-94805 Villejuif, France

**Keywords:** Infertility, Cost analysis, Childhood cancer survivors, Cancer treatments

## Abstract

**Purpose:**

This retrospective cohort study estimated the healthcare expenditure of infertility treatment in women who survived childhood cancer and analyzed associated factors using data from the French Childhood Cancer Survivor Study (FCCSS) cohort.

**Patients and methods:**

From 2578 women in the FCCSS cohort, 118 exposed who received infertility treatment, aged 18–42, were identified based on first recorded drug therapy and/or in vitro fertilization/intracytoplasmic sperm injection and/or medically assisted procreation between 2011 and 2018. Six hundred eight unexposed who had not undergone infertility treatment were selected using propensity score matching. Expenditure related to infertility treatment was estimated as the difference between cases’ expenditures and average expenditures of their controls yearly, over a maximum follow-up period of 4 years unexposed.

**Results:**

Average annual expenditure related to infertility treatment per patient over 4 years was 3043 euros (95% CI: 2240; 3845) (in euros 2025). Expenditure peaked during the first 2 years and among women aged 25–35. Patients treated with radiotherapy alone had higher average annual expenditure of 4531 euros (95% CI: 1781; 7281). In multivariate analysis, radiotherapy alone and chemotherapy alone were associated with increased annual expenditure of 4382 euros (95% CI: 1102.0; 7661.3) and 2018 euros (95% CI: 97.3; 3937.8), respectively, compared to patients without childhood radiotherapy or chemotherapy. Combined radiotherapy and chemotherapy increased infertility-related expenditure (€2132.5 (95% CI: −36.6; 4301.6), *p*: 0.054) compared with patients who received neither of these two treatments. The magnitude, trends of expenditure, and age at initiation of infertility treatment were consistent with the general population.

**Conclusion Implications for Cancer Survivors:**

The study shows that childhood cancer treatment modalities significantly influence expenditures related to infertility treatment in women, which are implicitly attributable to the adverse effects of these treatments,with radiotherapy being a key driver. These findings support developing public health strategies promoting more targeted paediatric cancer treatments to mitigate long-term costs, particularly those associated with infertility treatment.

**Supplementary Information:**

The online version contains supplementary material available at https://doi.org/10.1007/s11764-026-02032-2.

## Introduction

The registered age-standardized incidence rate of cancer in children aged 0–14 years rose from 124.0 to 140.6 per million person-years between 1980 and 2010 [1]. Between 1991 and 2010, the average annual age-standardized incidence in European children aged 0 to 14 years was 137.65 per million person-years, and increased significantly by 0.54% over the period [2]. At the same time, survival in children with cancer improved to a 5-year overall survival rate of over 80% [3]. Studies have shown that survivors of childhood cancer are at high risk of suffering from late sequelae induced by the disease and cancer treatment [4–8]. These sequelae can affect any organ. The genital organs are not spared, which can lead to infertility in adulthood.

The risk of clinical infertility in adulthood for female survivors of pediatric cancer is 1.48 (95% CI: 1.23; 1.78) times higher compared to siblings [9]. Similarly, a history of cancer is associated with a lower number of live births. The incidence rate ratio of live births compared to women of the same age who have not had cancer is 0.69 (confidence interval (95% CI: 0.67; 0.70) [10]. This reduction in fertility is mainly linked to treatments that can affect the gonads and the hypothalamo-hypophyseal axis which control fertility. Although younger patients have a greater number of oocytes and may therefore appear less vulnerable to gonadal damage than older patients, these treatments can still significantly impair reproductive function [11]. Chemotherapy can cause primary ovarian failure, reducing the chances of live birth [12–14]. Similarly, radiation therapy targeting the abdomen, pelvis, spine, brain or adjacent areas can damage reproductive organs and the pituitary gland [15]. Surgery for cancers of the reproductive tract and pelvic region can also damage nearby reproductive tissue, cause scarring and thus impair fertility [16]. In certain cancer situations where fertility is threatened, fertility preservation techniques may be considered [17]. For women, this can include cryopreservation of oocytes or embryos or ovarian tissue before undergoing medical treatment such as chemotherapy or surgery [17].

The burden of infertility can be particularly significant at the individual level, especially in this population with a long medical history [18]. Nevertheless, infertility should also be considered from a collective perspective as it is increasingly recognized as a public health issue, although its economic impact on the healthcare system remains insufficiently documented. Childhood cancer survivors are known to incur higher healthcare expenditures in adulthood compared to the general population or their siblings, largely due to a higher burden of comorbidities from all causes combined [6, 19]. In France, data from the French Childhood Cancer Survivor Study (FCCSS) estimated this excess expenditure at 3920 euros (95% CI: 3539; 4301) per year [6]. Given the increased risk of infertility in this population, and the longer time required to achieve pregnancy [9], documenting the expenditures associated with infertility treatment appears particularly relevant. Providing robust cost estimates can help policymakers better understand the budgetary implications and anticipate future needs in a context of limited healthcare resources, thereby highlighting the importance of the concept of opportunity cost [20]. In addition, such estimates provide essential baseline data for future cost-effectiveness analyses, which are crucial when evaluating alternative treatment strategies or potential changes in health policies.

The aim of this study was to estimate the healthcare expenditures of infertility treatment in women who survived childhood cancer, and to analyse the factors associated with it.

## Materials and methods

### FCCSS cohort

The FCCSS cohort was initially set up between 1985 and 1995 to assess the long-term effects of treatment in children who survived at least 5 years after the initial diagnosis of a solid cancer that occurred after 1945 [21]. In 2012, the cohort was expanded to include all children with pediatric cancer before 2000, to become the FCCSS cohort with 7670 subjects. A match to the National Health Data System (SNDS) was made in 2013 and again in 2020. The SNDS is a national medico-administrative database that contains all consumption and reimbursement data for hospital and outpatient care among health insurance beneficiaries in France [22]. It also contains information on patient-specifc administrative data, such as date of birth, gender, place of residence, and conditions for reimbursement of care (total or partial insurance coverage). In 2020, data from 5679 people (including both men and women) in the FCCSS cohort were linked to the SNDS. The postcode of residence enabled us to use the French Deprivation Index (Fdep13)[23]. The Fdep13 is an ecological measure that characterizes the socio-economic status of an individual according to his or her postal code of residence. It is defined at zip code level as the first component of a principal component analysis of the following four variables: median household income, percentage of graduates in the population aged over 15, percentage of workers in the active population and unemployment rate.

### Population

SNDS data, i.e., all data relating to the reimbursement of hospital and outpatient care for health insurance beneficiaries, are available for 2578 women in the FCCSS cohort since 2006 (date when SNDS data became available to researchers). Exposed women, i.e. women receiving treatment for infertility, were identified on the basis of the first recorded date of drug therapy (Supplemental Table 1) and/or an in vitro fertilization/intracytoplasmic sperm injection (IVF/ICSI) procedure (Supplemental Table 2) and/or a medically assisted procreation procedure (AMP) (Supplemental Table 2) between 2011 and 2018, without any treatment before. We chose to begin our analysis in 2011 because it is widely accepted that the SNDS data from 2011 onwards are preferable due to their greater comprehensiveness and stability, particularly with regard to hospital data, as well as the increased consistency of coding and reimbursement rules.

Only women aged between 18 and 42 at the date of first use of infertility-related care and residing in metropolitan France were included. Unexposed were defined as all women who had not undergone infertility treatment between 2011 and 2018.

### Matching method

Each exposed was matched to one or more unexposed using propensity score methods. Variables included in the propensity score model were selected a priori based on clinical relevance and prior literature [5, 6, 24]. Specifically, we included baseline characteristics measured before infertility treatment that were potential confounders, i.e., variables plausibly associated with both treatment exposure and subsequent healthcare expenditures.. These variables were:

- age in 2011 (start date of the study) categorized into twelve classes of 2-year intervals (19–20, 21–22, 23–24, 25–26, 27–28, 29–30, 31–32, 33–34, 35–36, 37–38, 39–40, 41–42)
- type of cancer treatment in four classes (radiotherapy, chemotherapy, radiotherapy and chemotherapy, neither radiotherapy nor chemotherapy)
- type of tumor (bone sarcoma, central nervous system tumor, gonadal tumor, kidney tumor, lymphoma, neuroblastoma, other solid cancers, retinoblastoma, soft tissue sarcoma, thyroid tumor)
- age at cancer diagnosis in five classes (0–1, 2–4, 5–9, 10–14 and >14 years)
- year of cancer diagnosis in two classes (1989 and under, 1990 and over)
- FDep13 quintile at start of infertility treatment (Quintile 1 (avantaged), 5 (disavantaged)).

These variables were used in Proc Psmatch, SAS® to calculate the propensity score (probability of being treated for infertility). In the propensity score, an exact match was made on type of tumor and age category in 2011. We also chose to use a non-replacement, non-optimal, “greedy” method, in which each exposed was matched sequentially with a maximum a priori fixed number (*k* = 10) of nearest unexposed [25, 26], using a calliper value (i.e., a threshold on the allowable distance between the propensity scores of the exposed and unexposed) at 0.4 (Supplemental material 3).

The follow-up start date of the unexposed was identical to that of the exposed to which they were matched. Exposed and unexposed were censored at the start of a full-term pregnancy. Those who died during or shortly after the 4-year follow-up were excluded because of the usually higher costs of care in the year preceding death, and the potential biases in the estimation of costs attributable to infertility treatment that this may generate.

### Economic analysis

Each exposed individual, indexed by $i=1,...,N_e$, was matched with $n_i$, unexposed individuals, indexed by $j=1,...,n_i$. Matching ensured comparability between exposed women and their unexposed. For both exposed and unexposed individuals, we collected all reimbursed healthcare expenditures incurred during the follow-up period. Expenditures included pharmacy, medical consultations, laboratory tests (biology), nursing care, midwifery, physiotherapy, dental care, medical transport, daily allowances, public and private hospitalizations.

Annual healthcare expenditures were computed for $t=1,...,T,with\;max\left(T\right)=4$. Let:

- $H{E}_{it}$ denote the annual healthcare expenditure for exposed individual i in year t
- $H{E}_{ijt}$ denote the annual healthcare expenditure for unexposed j matched to exposed individual i in year t
- For each exposed individual i, we defined the vector of excess healthcare expenditures:
  $$EH{E}_{i}=\left[\begin{array}{c}EH{E}_{i1}\\ \vdots \\ EH{E}_{iT}\end{array}\right]$$

where, for each year t:

$$EH{E}_{it}=H{E}_{it}-\frac{1}{{n}_{i}}\sum_{j=1}^{{n}_{i}}H{E}_{ijt}$$

This measure represents the difference between the healthcare expenditure of the exposed women, and the mean healthcare expenditure of her matched unexposed. We interpreted $EH{E}_{it}$ as the annual healthcare expenditure attributable to infertility treatment.

Excess expenditures were summarized as:

- Mean annual expenditure (euros) per exposed individual
- 95% confidence intervals
- Reported separately for each follow-up year

We also conducted this analysis for the year prior to infertility treatment (year 1) and the year following it (year 5) to ensure that the difference in expenditures between the exposed and their unexposed was not statistically significant.

This approach estimates individual-level excess healthcare costs within a matched counterfactual framework. By comparing exposed individuals with their matched controls over time, the analysis isolates expenditures plausibly attributable to infertility treatment.

As we explored the data, we realized that extreme values could have a negative impact on the robustness of the data. We therefore decided to remove expenditure values above the 95th percentile for both exposed and unexposed. Results are presented in euros 2025.

### Statistical analysis

Based on the results of a previous work [27], we chose to estimate the expenditure attributable to infertility treatment over a follow-up period of 4 years after the start of infertility treatment. We then described this expenditure according to the following characteristics of women:

o - time since the beginning of infertility treatment (Year 1, Year 2, Year 3, Year 4)
o - age at start of infertility treatment (18–24, 25–30, 31–35, 36–42)
o - type of cancer treatment in four classes (radiotherapy, chemotherapy, radiotherapy and chemotherapy, neither radiotherapy nor chemotherapy)
o - type of tumour (bone sarcoma, central nervous system tumor, gonadal tumor, kidney tumor, lymphoma, neuroblastoma, other solid cancers, retinoblastoma, soft tissue sarcoma, thyroid tumor)
o - age at cancer diagnosis in five classes (0–1, 2–4, 5–9, 10–14 and >14 years)
o - year of cancer diagnosis in two classes (1989 and under, 1990 and over)
o - Fdep13 quintile (Quintile 1–2 (avantaged), Quintile 3, Quintile 4–5(disavantaged)).

Finally, we examined the associations between all these variables using Pearson’s chi square test of independence, and performed a multivariate analysis to identify the factors associated with infertility treatment related expenditure. Given the presence of repeated data, a linear mixed model was used with a random intercept and a random slope [25].

To check the robustness of the multivariate analysis results, we varied the number of unexposed per exposed.

## Results

At the end of the matching process, 608 unexposed were matched to 118 exposed, representing a median of 5, a minimum of 1 and a maximum of 10 unexposed per exposed (Table 1). Table 1 shows the comparability of the variable distribution between the exposed and unexposed groups following propensity score matching. During the 4-year follow-up (or shortly after for death), no exposed individual was censored due to pregnancy or death.

**Table 1:** Descriptive table of exposed and unexposed after propensity score matching with a maximum number of 10 unexposed per exposed and a caliper of 0.4

Variable	Unexposed	Exposed	*p*-value	Std-D	V-ratio	Std-D (W)	V-ratio (W)	Std-D W < 0.1
**Logit of propensity score**			0.074	0.1771	1.1082	0.0394	1.0313	*
N	608	118						
Average (standard deviation)	−2.4 (0.6)	−2.3 (0.6)						
Median (Q1; Q3)	−2.3 (−2.8; −2.0)	−2.2 (−2.8; −1.9)						
(Min; max)	(−4.0; −0.9)	(−3.9; −0.9)						
**Age in 2011**			0.93	0.009	0.8584	0.0077	0.9734	*
Mean (SD)	608	118						
Median (Q1; Q3)	29.4 (5.5)	29.3 (5.1)						
(Min; max)	29.0 (25.0; 33.0)	30.0 (25.0; 33.0)						
**Age in 2011% (*n*)**			0.989					
19–20	4.1 (25)	3.4 (4)		0.038	0.8306	0	1	*
21–22	7.7 (47)	6.8 (8)		0.0367	0.8861	0	1	*
23–24	10.7 (65)	10.2 (12)		0.0171	0.9568	0	1	*
25–26	8.7 (53)	9.3 (11)		0.0211	1.0623	0	1	*
27–28	12.2 (74)	13.6 (16)		0.0415	1.0965	0	1	*
29–30	14.3 (87)	12.7 (15)		0.0467	0.9049	0	1	*
31–32	14.0 (85)	16.9 (20)		0.0822	1.1705	0	1	*
33–34	9.9 (60)	12.7 (15)		0.0899	1.2475	0	1	*
35–36	7.2 (44)	5.1 (6)		0.0896	0.7189	0	1	*
37–38	5.4 (33)	5.1 (6)		0.0154	0.9402	0	1	*
39–40	3.5 (21)	2.5 (3)		0.0535	0.743	0	1	*
41–42	2.3 (14)	1.7 (2)		0.0434	0.7407	0	1	*
**Type of cancer treatment % (*n*)**			0.705					
Neither radiotherapy nor chemotherapy	10.2 (62)	8.5 (10)		0.0592	0.847	0.0117	0.9649	*
Radiotherapy and chemotherapy	37.5 (228)	40.7 (48)		0.0652	1.0296	0.0534	1.0235	*
Radiotherapy alone	7.2 (44)	9.3 (11)		0.0757	1.2592	0.0534	1.1682	*
Chemotherapy alone	45.1 (274)	41.5 (49)		0.0715	0.9808	0.0754	0.9801	*
**Type of tumor % (*n*)**			0.999					
Bone sarcoma	11.7 (71)	11.0 (13)		0.0208	0.9505	0	1	*
Central nervous system tumor	9.7 (59)	10.2 (12)		0.0156	1.0426	0	1	*
Gonadal tumor	6.3 (38)	7.6 (9)		0.0486	0.9181	0	1	*
Kidney tumor	17.9 (109)	16.1 (19)		0.0189	1.0301	0	1	*
Lymphoma	18.8 (114)	19.5 (23)		0.0282	0.9483	0	1	*
Neuroblastoma	16.3 (99)	15.3 (18)		0.0005	1.0018	0	1	*
Other solid cancers	5.9 (36)	5.9 (7)		0.0465	1.2241	0	1	*
Retinoblastoma	4.1 (25)	5.1 (6)		0.0145	0.9584	0	1	*
Soft tissue sarcoma	8.9 (54)	8.5 (10)		0.0434	1.7114	0	1	*
Thyroid tumor	0.5 (3)	0.8 (1)		0.0434	1.7114	0	1	*
**Age at cancer diagnosis % (*n*)**			0.958					
0–1	20.1 (122)	19.5 (23)		0.0144	0.9784	0.0119	1.0188	*
2–4	21.4 (130)	20.3 (24)		0.0257	0.9639	0.0229	0.9676	*
5–9	25.8 (157)	23.7 (28)		0.0485	0.9449	0.0401	0.9537	*
10–14	22.0 (134)	24.6 (29)		0.06	1.0788	0.0361	1.0451	*
>14	10.7 (65)	11.9 (14)		0.0371	1.0952	0.0209	1.0511	*
**Year of diagnosis % (*n*)**			0.811					
1989 and under	49.5 (301)	48.3 (57)		0.024	0.9989	0.0109	0.9994	*
1990 and over	50.5 (307)	51.7 (61)		0.024	0.9989	0.0109	0.9994	*
**Quintile FDEP % (*n*)**			0.931					
1 (most advantaged)	21.9 (133)	20.3 (24)		0.0376	0.9481	0.0385	0.947	*
2	18.4 (112)	21.2 (25)		0.0694	1.1111	0.0824	1.1361	*
3	22.9 (139)	20.3 (24)		0.0613	0.9187	0.0317	0.9553	*
4	17.6 (107)	18.6 (22)		0.0271	1.046	0.0072	1.0117	*
5 (most disadvantaged)	19.2 (117)	19.5 (23)		0.0063	1.0098	0.0174	0.9743	*

Caliper: threshold on the allowable distance between the propensity scores of the exposed and unexposed

FDEP: tercile of social deprivation index (indice de désavantage social) is an ecological measure that characterizes the socio-economic environment in which individuals in a given geographic area live at a given time, based on the percentage of workers in the labour force, the percentage of high school graduates aged 15 and over, the percentage of unemployed in the labour force, and median household income

*Std-D* standardized difference, *V-ratio* variance ratio, *Std-D (W)* weighted standardized difference, *V-ratio (W)*weighted variance ratio

### Descriptive results of infertility treatment related expenditure

The average annual expenditure over 4 years related to infertility treatment per patient who survived pediatric cancer was 3043 euros (95% CI: 2240; 3845) (Table 2). The difference in expenses between exposed and unexposed in the year preceding infertility treatment and year 5 was 415 (−246; 1077) and 707 euros (−843; 2256), which was not statistically significant. Expenditure attributable to infertility treatment occurred mainly during the first 2 years, decreasing during years 3 and 4. Most women seek infertility treatment between ages 25 and 35 (25–30 (*n* = 51 (43%): €2848 (95% CI: 1813; 3883)); 31–35 (*n* = 41 (35%): €3395 (95% CI: 1958; 4832))), with lower annual expenditure when treatment was initiated between ages 36 and 42 (€1259 (95% CI: −558; 3076)). Radiotherapy alone was associated with higher average annual expenditure of 4531 euros (95% CI: 1781; 7281). Chemotherapy alone, or with radiotherapy, was associated with infertility treatment related expenditure comparable to average annual expense. Annual expenditure for women with tumors of central nervous system, thyroid or retinoblastoma were far from average. Average annual expenditure was lower among more advantaged women.

**Table 2:** Descriptive table of average infertility treatment related expenditure per year and per exposed in euros 2025

Variables	N_exposed_ (%)	N_observations_(%)	Mean (95% CI)
**Total**	118	335	3043 (2240; 3845)
**Time since start of follow up**			
Year before start of follow up	116 (98)	116 (98)	415 (−246; 1077)
Year 1	118 (100)	118 (100)	4051 (3139; 4962)
Year 2	87 (74)	87 (74)	3979 (2772; 5187)
Year 3	72 (61)	72 (61)	1740 (713; 2766)
Year 4	58 (49)	58 (49)	1204 (41; 2366)
Year 5	45 (38)	45 (38)	707 (−843; 2256)
**Age at start of infertility treatment**			
18–24	5 (4)	18 (5)	3342 (596; 6089)
25–30	51 (43)	131 (39)	2848 (1813; 3883)
31–35	41 (35)	122 (36)	3395 (1958; 4832)
36–42	21 (18)	64 (19)	1259 (−558; 3076)
**Type of cancer treatment**			
Neither radiotherapy nor chemotherapy	10 (8)	18 (5)	1423 (−1; 2847)
Radiotherapy and chemotherapy	48 (41)	138 (41)	2669 (1339; 3999)
Radiotherapy alone	11 (9)	29 (9)	4531 (1781; 7281)
Chemotherapy alone	49 (42)	150 (45)	2674 (1588; 3760)
**Type of tumor**			
Bone sarcoma	13 (11)	40 (12)	3275 (582; 5968)
Central nervous system tumor	12 (10)	32 (10)	−46 (−2346; 2255)
Gonal tumor	9 (8)	24 (7)	3088 (1636; 4540)
Kidney tumors	19 (16)	52 (16)	3268 (1263; 5272)
Lymphoma	23 (19)	65 (19)	2743 (1472; 4013)
Neuroblastoma	18 (15)	51 (15)	2362 (667; 4057)
Other solid cancer	7 (6)	20 (6)	3438 (1468; 5407)
Retinoblastoma	6 (5)	14 (4)	4312 (−284; 8908)
Soft tissue sarcomas	10 (8)	33 (10)	3166 (−573; 6905)
Thyroid tumor	1 (1)	4 (1)	4775 (4775; 4775)
**Year of cancer diagnosis**			
1989 and under	57 (48)	171 (51)	2306 (1117; 3495)
1990 and over	61 (52)	164 (49)	3256 (2279; 4232)
**Age at cancer diagnosis**			
0–1	23 (19)	64 (19)	2794 (843; 4745)
2–4	24 (20)	70 (21)	1823 (619; 3026)
5–9	28 (24)	80 (24)	3061 (1499; 4624)
10–14	29 (25)	80 (24)	3290 (1448; 5132)
> 14	14 (12)	41 (12)	2764 (708; 4819)
**Quintile FDEP 13**			
Quintile 1–2 (advantaged)	49 (42)	134 (40)	2274 (1024; 3524)
Quintile 3	24 (20)	68 (20)	3187 (1412; 4961)
Quintile 4–5 (disadvantaged)	45 (38)	133 (40)	3032 (1850; 4214)

FDEP: tercile of social deprivation index (indice de désavantage social) is an ecological measure that characterises the socioeconomic environment in which individuals in a given geographic area live at a given time, based on the percentage of workers in the labour force, the percentage of high school graduates aged 15 and over, the percentage of unemployed in the labour force, and median household income

Due to associations between age at start of infertility treatment and both age at cancer diagnosis (Supplemental Table 4), and year at cancer diagnosis (Supplemental Table 5), these variables were excluded from multivariate analysis to avoid multicollinearity. Tumor type was excluded due to its association with cancer treatment type (Supplemental Table 6).

### Multivariate analysis—factors associated with infertility treatment related expenditure

The primary finding was that the type of cancer treatment was significantly associated with higher infertility treatment related expenditure (Table 3). Radiotherapy alone and chemotherapy alone increased annual expenditure by 4382 euros (95% CI: 1102.0; 7661.3) and 2018 euros (95% CI: 97.3; 3937.8) respectively, compared to a patient with childhood cancer who would not have undergone radiotherapy or chemotherapy during childhood. The combination of radiotherapy and chemotherapy tend to increase infertility treatment related expenditure (€2132.5 (95% CI: −36.6; 4301.6.0), *p*: 0.054) compared with a patient who had not undergone radiotherapy or chemotherapy during childhood. There was a negative significant interaction between radiotherapy and chemotherapy (*p* = 0.027).

**Table 3:** Results of multivariate analysis of average annual infertility treatment related expenditure per exposed in euros

	Coefficient (95% CI)	*P* value
**Time since start of follow up**		<0.001
Year 1	2634.9 (1375.5; 3894.2)	<0.001
Year 2	2476.6 (1157.1; 3796.2)	<0.001
Year 3	259.5 (−714.0; 1233.1)	0.599
Year 4	Ref	
**Age at start of infertility treatment**		0.469
18–24	Ref	
25–30	391.1 (−2961.9; 3744.1)	0.818
31–35	288.8 (−3185.0; 3762.6)	0.87
36–42	−1108.2 (−4666.7; 2450.3)	0.539
**Type of cancer treatment**		0.033
Neither radiotherapy nor chemotherapy	Ref	
Radiotherapy and chemotherapy	2132.5 (−36.6; 4301.6)	0.054
Radiotherapy alone	4381.6 (1102.0; 7661.3)	0.009
Chemotherapy alone	2017.5 (97.3; 3937.8)	0.04
**Quintile FDEP 13**		0.72
Quintile 1–2 advantaged	Ref	
Quintile 3	262.2 (−1862.8; 2387.2)	0.807
Quintile 4–5 disadvantaged	636.7 (−920.6; 2194.0)	0.42
**Model statistics**		
Number of subjects	118	
Number of observations (min–max per women)	335 (1–4)	
Mean of observations per women (95% CI)	2.8 (2.6–3.1)	

FDEP: tercile of social deprivation index (indice de désavantage social) is an ecological measure that characterises the socio-economic environment in which individuals in a given geographic area live at a given time, based on the percentage of workers in the labour force, the percentage of high school graduates aged 15 and over, the percentage of unemployed in the labour force, and median household income

Reading grid: women who started infertility treatment at 31–35 had an average annual expenditure of 421.3 euros more than women who started treatment at 18–24

There was a negative significant interaction between radiotherapy and chemotherapy (*p* = 0.027)

The first 2 years of treatment were significantly associated with higher infertility treatment related expenditure (reference: Year 4), whereas the age at which infertility treatment began and Fdep 13 were not.

An initial sensitivity analysis carried out to check the robustness of the results of the multivariate model revealed no variation in results according to the number of unexposed (Supplemental Table 7).

A second sensitivity analysis was carried out to assess the impact of associated variables in the multivariate analysis model. Age at start of infertility treatment was successively replaced by year of cancer diagnosis and age at cancer diagnosis, with no substantial impact on the results, except for strengthening the significance of the association between the childhood cancer treatment type and infertility treatment expenses (Supplemental Table 8).

## Discussion

This study was based on the FCCSS cohort to assess the healthcare expenditure of infertility treatment in women who survived pediatric cancer. It included 118 exposed seeking infertility treatment between 2011 and 2018. These exposed were matched to 608 unexposed. The main result of this study demonstrates an association between radiotherapy and/or chemotherapy in childhood and increased expenditure of infertility management in adulthood. In addition, we also find that the magnitude and trends of expenditure, and the age at initiation of infertility treatment in women with a history of pediatric cancer are consistent with those observed in the general population.

### Healthcare expenditure of infertility treatment

This is the first study examining the healthcare expenditure of infertility treatment in women who survived pediatric cancer. For comparison, a recent French study in the general population showed that average annual expenditure per woman for infertility treatment (556 women followed over 3.5 years) was 2018 euros (95% CI: 1609; 2428) [27]. Our result aligns with expectations of higher infertility treatment expenditure in female cancer survivors (3043 euros (95% CI: 2240; 3845)), though the economic significance remains uncertain [28]. Infertility in this population mainly requires gonadotrophins and assisted reproductive technologies, which has the effect of pulling up average expenditures. In this population, the probability of becoming pregnant is lower than in the general population, as shown in the Childhood Cancer Survivor Study (CCSS) Cohort. The CCSS cohort was implemented at 26 participating clinical research centers in the USA and Canada, including survivors of childhood cancer diagnosed before age 21 between 1970 and 1986. It includes a randomly selected subset of survivors’ siblings as a control group and 5149 women aged 15–44. The pregnancy probability in women with pediatric cancer is lower than their sisters ((RR) of 0.81 (95% CI: 0.73; 0.90; *p* < 0.001)) [29], leading to more specialized care with higher expenditures. Regarding expenditure evolution since treatment start (Year 1: 4051 (95% CI: 3139; 4962), Year 2: 3979 (95% CI: 2772; 5187), Year 3: 1740 (95% CI 713; 2766), Year 4: 1204 (95% CI: 41; 2366)), results show a sharp increase at treatment initiation, with gradual decrease thereafter [27].

The age for seeking infertility treatment falls between 25 and 35, with mean age 31.8 (95% CI 31.0; 32.6). This matches a 2020 French study using Echantillon Généraliste des Bénéficiaires (EGB) data, which included over 100,000 prevalent women yearly, reporting average age 33 for initiating treatment [30]. These findings align with other studies on infertility drug treatment timing [31, 32]. Clomiphene citrate is mainly used by patients aged 25–30 and 31–35, with frequency per 1000 women of 6.59 (95% CI: 6.21; 6.99) and 5.17 (95% CI: 4.80; 5.57), respectively. Gonadotropins are mainly used by women aged 31–35, with frequency per 1000 women of 6.18 (95% CI: 5.78; 6.61) [32].

In multivariate analysis, no association was found between the social deprivation index and the average annual expenditure of infertility treatment. This result is not surprising, as these treatments are fully covered in France, meaning their expenditure does not affect access. In other words, individuals from disadvantaged backgrounds do not appear to use these treatments differently from those from more privileged settings. This is particularly noteworthy given the strong association between a history of childhood cancer and socioeconomic disadvantage in adulthood [33].

The average expenditure per woman per year for infertility treatment varies by cancer type. For central nervous system tumors, the expenditure is close to zero, which may reflect the severity of long-term sequelae and the associated healthcare costs. Women who underwent infertility treatment may have been those with less severe sequelae—our exposed—while unexposed may have had higher healthcare expenditures. This could lead to a potential underestimation of infertility treatment related costs. It is possible that a similar pattern exists for other types of cancer and is modulated by the type of treatment, the location, and the dose of the treatment.

Concerning the time horizon for this study, we chose a duration of 4 years based on findings from a previous analysis of the economic burden of infertility treatment in the general population [27]. That study showed that women treated for infertility incurred higher costs beyond 4 years compared to untreated women; however, this difference was neither statistically nor economically significant. We confirmed retrospectively that this time horizon was appropriate as the difference in expenses between the exposed and the unexposed during the year before treatment (year −1) and the year after (year 5) were not significant, 415 (−246; 1077) and 707 euros (−843; 2256), respectively.

### Radiotherapy associated with increased expenditures

The main finding from the multivariate analysis indicated a significant association between the type of childhood cancer treatment and the infertility treatment related expenditures incurred in adulthood, thus confirming our original hypothesis. Specifically, compared with neither radiotherapy nor chemotherapy in childhood, radiotherapy alone, chemotherapy alone and combined radiotherapy and chemotherapy, increased the expenditures of managing infertility in adulthood by 4381.6 euros (95% CI: 1102; 7661.3; *p* = 0.09), 2017.5 euros (95% CI: 97.3; 3937.8; *p* = 0.04) and 2132.5 euros (−36.6; 4301.6; *p* = 0.054), respectively. The association of combined radiotherapy and chemotherapy was at the threshold of significance in the main model likely reflecting the limited sample size. These findings are supported by existing evidence showing a strong association between both radiotherapy and chemotherapy and the risk of premature ovarian failure, which subsequently increases the likelihood of infertility treatment [11]. Indeed, the prevalence of premature ovarian failure (whose definition may vary from one study to another) was 10.9% and 9.1% in the St. Jude Lifetime Cohort Study and the CCSS, respectively, compared with an estimated 1% in the general population [34–36]. The degree of chemotherapy-induced damage varies depending on drug type, dose, and the patient-specific factors such as age and sex [12–14], while radiotherapy-related damage depends on the treatment field, total dose and fractionation schedule [15].

Furthermore, these results align with a recent study assessing excess long-term healthcare expenditure among individuals in the FCCSS cohort. This study included 5353 people from the FCCSS cohort matched with 382,757 controls from the EGB by age, sex and region of residence, and reported an excess annual expenditure of 1910 euros for those treated with radiotherapy compared to those who had neither chemotherapy nor radiotherapy (*p* = 0.01) [6, 24].

The negative interaction between chemotherapy and radiotherapy that we observed has been highlighted in literature focusing on long-term sequelae of childhood cancer treatments. However, proposed biological explanations for this interaction remain unconfirmed [37, 38].

### Strengths and limitations

The main strength of this study lies in its database. Few prospective cohorts of childhood cancer patients have sufficient hindsight to analyze infertility treatment use [21, 39]. The prospective nature of the FCCSS limits classification bias on initial patient characteristics.

The main limitation lies in the sample size, which prevented incorporating tumor type into the multivariate model. Tumor type is likely associated with infertility treatement expenditure, as there is a gradation of infertility risk from low to high depending on cancer type [11]. This risk is partly mediated by treatment type, a variable we introduced into the model and found significant.

A second limitation is the non-inclusion of radiotherapy dose and site in the propensity score model, although this dose can vary across treatment centers due to differences in protocols, expertise, and technology. In some cases (lymphoma, sarcoma, etc.), the site of the disease may be different, so patients with the same diagnosis may receive radiation therapy to different areas, which can have different effects on fertility. The same applies to the type of chemotherapy and the dose. Such variability may affect long-term outcomes, including fertility. Therefore, the cost differential between exposed and unexposed may be under- or overestimated. Including these different parameters would have enhanced accuracy in estimating its effect. However, this data was unavailable but may be considered in future studies.

The third limitation is not accounting for fertility preservation. The risk of developing a second cancer 30 years after the first is estimated at 10% [40]. We could not distinguish whether infertility treatment related to fertility preservation for a second cancer or to addressing infertility from childhood cancer. This likely caused slight underestimation of childhood cancer-related infertility management expenditure, given the small number of individuals concerned, but likely did not impact factors associated with these expenditures.

Finally, our results are specific to the French healthcare system, which is characterized by near-universal coverage, broad access to care, and reimbursement and coding procedures unique to its organization. Consequently, the magnitude of infertility treatment expenditures may differ in other contexts. However, the treatments and care modalities studied are not unique to France, and there is no reason to believe that the factors influencing these expenditures would differ elsewhere. Nevertheless, further studies in other national contexts are needed to confirm the external generalizability of our results, particularly in healthcare systems with substantially different access to care, financing mechanisms, or structural organization structures.

## Conclusions

Childhood cancer treatment type significantly influenced infertility treatment related expenditures in women. Radiotherapy—especially when used alone—was associated with higher expenditures. These findings underscore the need for public health strategies promoting more targeted paediatric cancer treatments to reduce long-term healthcare expenditures, especially those associated with infertility treatment.

## Supplementary Information

Below is the link to the electronic supplementary material.

ESM1 (DOCX 309 KB)

## Data Availability

The data of the FCCSS which are used in this analysis are available upon request from Brice Fresneau, brice.fresneau@gustaveroussy.fr.
