# Supplementary material for: Infertility costs and associated factors in childhood cancer survivors: the role of radiotherapy
Source: J Cancer Surviv. 2026 May 8;20(Suppl 2):2009–19. doi: 10.1007/s11764-026-02032-2 (PMC13744461; doi:10.1007/s11764-026-02032-2)
Supplement: Supplementary file 1 [file 11764_2026_2032_MOESM1_ESM.docx]

Supplemental table 1 : List of pharmaceutical treatments used to identify women treated for infertility

Supplemental table 2 : Acts used to identify women treated for infertility

| **Type of act** | | **Code** | **Name** |
| --- | --- | --- | --- |
| Biology | bio_prs_ide | 60 | in vitro fertilization |
|  |  | 61 | Intracytoplasmic Sperm Injection |
| Technical acts | cam_prs_ide | JJFC011 | Laparoscopic oocyte retrieval from one or both ovaries |
|  |  | JJFJ001 | Transvaginal, ultrasound-guided oocyte retrieval from one or both ovaries |
|  |  | JKHD002 | Cervical mucus sampling and examination, without sperm motility test |
|  |  | JSEC001 | Laparoscopic intratubal embryo transfer |
|  |  | JSED001 | Intrauterine vaginal embryo transfer |
|  |  | JSLD001 | Intrauterine artificial insemination |
|  |  | JSLD002 | Intracervical artificial insemination |

Supplemental material 3 : Caliper choice

The propensity score was calculated using SAS® and these variables were used in the Proc Psmatch procedure to calculate the propensity score (probability of being treated) in exposed and unexposed based on a logistic regression. In the propensity score, exact matching was performed on tumor type and age category in 2011. Thus, the matching of 1 to k unexposed to their closest exposed was done by group type of initial tumor and age in 2011. The Psmatch procedure allows us to simultaneously manage exact matching and the calculation of propensity scores on other variables.

Change in the average standardized differences of the models and the number of observations according to caliper, order, and number of controls per case

For the final sample selection, two options in Proc Psmatch influenced the number of exposed and unexposed selected: the maximum number of unexposed (k) per exposed and the caliper.

The caliper is a parameter that sets the maximum distance (or proximity) at which a unexposed can be from a exposed in order for there to be a match. In other words, the difference between the propensity scores for pairs of individuals in the two groups had to be less than or equal to the caliper times the pooled estimate of the common standard deviation of the propensity scores. By default, the caliper is set to 0.25 in Proc Psmatch, although a caliper of 0.20 seems ideal in most situations. In our study, since the patients were already similar due to their clinical history and the exact matching variables included in the model, we chose to vary the caliper between 0.25, 0.4, and 2, and we varied the maximum number of unexposed per exposed between 3 and 20.

For each k-caliper pair, the mean standardized differences and the number of observations (in person-years) that the model allowed to be included were plotted on a graph (ci bellow). This graph was used to select the ideal number of unexposed per exposed based on the caliper. The mean standardized difference was defined as the absolute difference in the mean propensity score for each group divided by a pooled estimate of the variance in propensity scores. For each model, we considered a mean standardized difference of less than 0.10 to be acceptable.

Once the ideal number of unexposed per exposed had been identified for each of the three calipers (0.25, 0.4, and 2), we specifically analyzed each of the variables in the three identified models, thereby enabling us to evaluate the quality of the models and retain the most appropriate one. Thus, for each variable in each of the three models, we analyzed the weighted standardized differences, for which some authors recommend an upper limit of 0.25, while others recommend 0.10. We also analyzed the weighted variance ratio, which should tend toward 1. As we can see in Table 1, the weighted standardized differences are consistently less than 0.1, which indicates good matching.

Supplemental table 4 : Association between age at start of infertility treatment and age at cancer diagnosis using Person’s chi square test of independence

| Age at cancer  diagnosis | **0-1** | **2-4** | **5-9** | **10-14** | **>14** | **Total** | **p-val** |
| --- | --- | --- | --- | --- | --- | --- | --- |
|  | **% (N)** |  |  |  | **% (N)** | **% (N)** |  |
| **Total (line %)** | 19.1 (64) | 20.9 (70) | 23.9 (80) | 23.9 (80) | 12.2 (41) | **100.0 (335)** |  |
| **Age at start of**  **infertility treatment** |  |  |  |  |  |  | **<0.001** |
| 18-24 | 15.6 (10) | 5.7 (4) | 5.0 (4) | 0.0 (0) | 0.0 (0) | 6.3 (18) |  |
| 25-30 | 37.5 (24) | 52.9 (37) | 38.8 (31) | 36.3 (29) | 24.4 (10) | 38.6 (131) |  |
| 31-35 | 34.4 (22) | 31.4 (22) | 26.3 (21) | 47.5 (38) | 46.3 (19) | 37.2 (122) |  |
| 36-42 | 12.5 (8) | 10.0 (7) | 30.0 (24) | 16.3 (13) | 29.3 (12) | 18.9 (64) |  |
| Total | 100 (64) | 100 (70) | 100 (80) | 100 (84) | 100 (41) | 100.0 (335) |  |

In the context of Person's chi-square test of independence, a p-value of less than 0.05 means that there is an association between the two variables being tested.

Supplemental table 5 : Association between age at start of infertility treatment and year of cancer diagnosis

| Year of cancer diagnosis | **1989 and under** | **1990 and more** | **Total** | **p-val** |
| --- | --- | --- | --- | --- |
|  | **% (N)** | **% (N)** | **% (N)** |  |
| **Total (line %)** | **51.0 (171)** | **49.0 (164)** | **100.0 (335)** |  |
| **Age at start of infertility treatment** |  |  |  | **<0.001** |
| 18-24 | 0.0 (0) | 10.8 (18) | 5.4 (18) |  |
| 25-30 | 25.7 (44) | 53.0 (87) | 39.1 (131) |  |
| 31-35 | 42.1 (72) | 30.5 (50) | 36.4 (122) |  |
| 36-42 | 32.2 (55) | 5.5 (9) | 19.1 (64) |  |
| Total | 100.0 (171) | 100.0 (164) | 100.0 (335) |  |

In the context of Person's chi-square test of independence, a p-value of less than 0.05 means that there is an association between the two variables being tested.

Supplemental table 6 : Association between tumor class and Type of cancer treatment

| Type of cancer treatment | **Neither radiotherapy nor chemotherapy** | **Radiotherapy and chemotherapy** | **Radiotherapy alone** | **Chemotherapy alone** | **Total** | **p-value** |
| --- | --- | --- | --- | --- | --- | --- |
|  | **% (N)** | **% (N)** | **% (N)** | **% (N)** | **% (N)** |  |
| **Total (%line)** | **5.4 (18)** | **41.2 (138)** | **8.7 (29)** | **44.8 (150)** | **335** |  |
| **Tumor class** |  |  |  |  |  | **0.0427** |
| Bone sarcoma | 0.0 (0) | 5.5 (8) | 0.0 (0) | 21.3 (32) | 11.9 (40) |  |
| Central nervous system tumor | 16.7 (3) | 12.7 (18) | 37.9 (11) | 0.0 (0) | 9.6(32) |  |
| Gonadal tumor | 27.8 (5) | 0.0 (0) | 3.5 (1) | 12.0 (18) | 7.2 (24) |  |
| Kidney tumor | 5.6 (1) | 23.9 (34) | 13.8 (4) | 8.7 (13) | 15.5 (52) |  |
| Lymphoma | 0.0 (0) | 28.2 (40) | 0.0 (0) | 16.7 (25) | 19.4 (65) |  |
| Neuroblastoma | 22.2 (4) | 9.9 (14) | 3.5 (1) | 21.3 (32) | 15.2 (51) |  |
| Other solid cancers | 27.8 (5) | 0.0 (0) | 0.0 (0) | 10.0 (15) | 6.0  (20) |  |
| Retinoblastoma | 0.0 (0) | 3.5 (5) | 27.6 (8) | 0.7 (1) | 4.2 (14) |  |
| Soft tissue sarcoma | 0.0 (0) | 13.4 (19) | 0.0 (0) | 9.3 (14) | 9.9 (33) |  |
| Thyroid tumor | 0.0 (0) | 0.0 (0) | 13.8 (4) | 0.0 (0) | 1.2 (4) |  |
| Total | 100.0 (18) | 100.0 (138) | 100.0 (29) | 100.0 (150) | 100.0 (335) |  |

In the context of Person's chi-square test of independence, a p-value of less than 0.05 means that there is an association between the two variables being tested.

Supplemental table 7 : Robustness analysis of the multivariate model by varying the maximum a priori fixed number (k) of nearest unexposeds.

| **Variable** | **Coef (95%CI)** | **p-val** | **Coef (95%CI)** | **p-val** | **Coef (95%CI)** | **p-val** |
| --- | --- | --- | --- | --- | --- | --- |
| **Total** | 2,584 (1,674 ; 3,495) |  | 2,834 (1,942 ; 3,722) |  | 3,083 (2,274 ; 3,892) |  |
| **Time since start of follow up** |  | **<0.001** |  | **<0.001** |  | **<0.001** |
| Year 1 | 2,536.5 (1,342.2 ; 3,730.8) | <0.001 | 2,712.3 (1,491.0 ; 3,933.7) | <0.001 | 2,735.9 (1,456.7 ; 4,015.1) | <0.001 |
| Year 2 | 2,410.3 (1,085.7 ; 3,734.9) | <0.001 | 2,732.8 (1,385.4 ; 4,080.2) | <0.001 | 2,672.3 (1,328.9 ; 4,015.7) | <0.001 |
| Year 3 | 80.0 (-989.0 ; 1,149.1) | 0.882 | 399.7 (-620.6 ; 1,419.9) | 0.44 | 477.1 (-481.0 ; 1,435.2) | 0.326 |
| Year 4 | Ref |  | Ref |  | Ref |  |
| **Age at start of infertility treatment** |  | **0.357** |  | **0.451** |  | **0.441** |
| 18-24 | Ref |  | Ref |  | Ref |  |
| 25-30 | 3,23.9 (-2,734.0 ; 3,381.8) | 0.834 | 776.1 (-2,420.7 ; 3,972.8) | 0.632 | 486.6 (-2,826.5 ; 3,799.8) | 0.772 |
| 31-35 | -93.9 (-3,330.3 ; 3,142.6) | 0.954 | 135.4 (-3,218.5 ; 3,489.3) | 0.936 | 320.9 (-3,130.1 ; 3,771.9) | 0.854 |
| 36-42 | -1,599.6 (-4,959.4 ; 1,760.2) | 0.348 | -938.2 (-4,465.1 ; 2,588.7) | 0.6 | -1,059.7 (-4,570.0 ; 2,450.5) | 0.551 |
| **Type of treatment** |  | **0.039** |  | **0.043** |  | **0.033** |
| No radiotherapy or chemotherapy | Ref |  | Ref |  | Ref |  |
| Radiotherapy, chemotherapy | 2,098.5 (-63.7 ; 4,260.7) | 0.057 | 1,851.1 (-275.0 ; 3,977.3) | 0.087 | 2,186.0 (-24.5 ; 4,396.5) | 0.053 |
| Radiotherapy | 4,291.5 (993.1 ; 7,589.8) | 0.011 | 4,387.6 (1,079.8 ; 7,695.4) | 0.01 | 4,466.7 (1,135.4 ; 7,798.0) | 0.009 |
| Chemotherapy | 1,741.6 (-169.4 ; 3,652.6) | 0.074 | 1,567.3 (-289.9 ; 3,424.5) | 0.097 | 2,015.2 (85.2 ; 3,945.3) | 0.041 |
| **Quintile FDep13** |  | **0.742** |  | **0.838** |  | **0.807** |
| Quintile 1-2 | Ref |  | Ref |  | Ref |  |
| Quintile 3 | 172.2 (-2,073.2 ; 2,417.6) | 0.88 | 435.9 (-1,724.7 ; 2,596.5) | 0.69 | 240.1 (-1,902.4 ; 2,382.6) | 0.825 |
| Quintile 4-5 | 627.5 (-1,006.9 ; 2,261.9) | 0.449 | 459.2 (-1,166.9 ; 2,085.2) | 0.577 | 513.5 (-1,040.7 ; 2,067.6) | 0.514 |
| **Model statistics** |  |  |  |  |  |  |
| Number of subjects | 119 |  | 119 |  | 119 |  |
| Number of observations | 336 |  | 339 |  | 339 |  |
| PSMATCH Infos | K=3, |  | K=4, |  | K=9, |  |

Supplemental table 7 : Robustness analysis of the multivariate model by varying the maximum a priori fixed number (k) of nearest unexposeds (continued).

| **Variable** | **Coef (95%CI)** | **p-val** | **Coef (95%CI)** | **p-val** |  | **Coef (95%CI)** | **p-val** | **Coef (95%CI)** | **p-val** |
| --- | --- | --- | --- | --- | --- | --- | --- | --- | --- |
| **Total** | 3,042 (2,240 ; 3,845) |  | 3,044 (2,244 ; 3,844) |  |  | 3,064 (2,271 ; 3,856) |  | 3,073 (2,281 ; 3,865) |  |
| **Time since start of follow up** |  | **<0.001** |  | **<0.001** |  |  | **<0.001** |  | **<0.001** |
| Year 1 | 2,634.9 (1,375.5 ; 3,894.2) | <0.001 | 2,676.2 (1,423.8 ; 3,928.7) | <0.001 |  | 2,705.6 (1,455.1 ; 3,956.0) | <0.001 | 2,728.2 (1,480.0 ; 3,976.3) | <0.001 |
| Year 2 | 2,476.6 (1,157.1 ; 3,796.2) | <0.001 | 2,522.7 (1,207.6 ; 3,837.7) | <0.001 |  | 2,525.5 (1,209.0 ; 3,842.0) | <0.001 | 2,518.2 (1,203.0 ; 3,833.4) | <0.001 |
| Year 3 | 259.5 (-714.0 ; 1,233.1) | 0.599 | 319.7 (-641.0 ; 1,280.4) | 0.511 |  | 302.9 (-655.3 ; 1,261.2) | 0.533 | 295.2 (-664.2 ; 1,254.5) | 0.544 |
| Year 4 | Ref |  | Ref |  |  | Ref |  | Ref |  |
| **Age at start of infertility treatment** |  | **0.469** |  | **0.476** |  |  | **0.402** |  | **0.398** |
| 18-24 | Ref |  | Ref |  |  | Ref |  | Ref |  |
| 25-30 | 391.1 (-2,961.9 ; 3,744.1) | 0.818 | 637.1 (-2,832.0 ; 4,106.2) | 0.717 |  | 528.4 (-2,915.9 ; 3,972.8) | 0.762 | 535.1 (-2,911.2 ; 3,981.5) | 0.759 |
| 31-35 | 288.8 (-3,185.0 ; 3,762.6) | 0.87 | 513.4 (-3,064.6 ; 4,091.5) | 0.777 |  | 363.3 (-3,192.0 ; 3,918.7) | 0.84 | 361.4 (-3,195.7 ; 3,918.4) | 0.841 |
| 36-42 | -1,108.2 (-4,666.7 ; 2,450.3) | 0.539 | -820.7 (-4,470.4 ; 2,829.0) | 0.657 |  | -1,042.0 (-4,654.9 ; 2,570.9) | 0.569 | -1,043.6 (-4,658.3 ; 2571.1) | 0.569 |
| **Type of treatment** |  | **0.033** |  | **0.031** |  |  | **0.034** |  | **0.033** |
| No radiotherapy or chemotherapy | Ref |  | Ref |  |  | Ref |  | Ref |  |
| Radiotherapy, chemotherapy | 2,132.5 (-36.6 ; 4,301.6) | 0.054 | 2,109.4 (-56.5 ; 4,275.3) | 0.056 |  | 2,188.1 (19.2 ; 4,357.0) | 0.048 | 2,198.5 (30.0 ; 4,367.0) | 0.047 |
| Radiotherapy | 4,381.6 (1,102.0 ; 7,661.3) | 0.009 | 4,420.4 (1,147.5 ; 7,693.4) | 0.009 |  | 4,306.7 (1,051.9 ; 7,561.5) | 0.01 | 4,310.0 (1,056.3 ; 7,563.7) | 0.01 |
| Chemotherapy | 2,017.5 (97.3 ; 3,937.8) | 0.04 | 1,994.8 (78.6 ; 3,910.9) | 0.041 |  | 2,048.5 (110.1 ; 3,986.9) | 0.039 | 2,074.7 (137.4 ; 4,011.9) | 0.036 |
| **Quintile FDep13** |  | **0.72** |  | **0.709** |  |  | **0.782** |  | **0.769** |
| Quintile 1-2 | Ref |  | Ref |  |  | Ref |  | Ref |  |
| Quintile 3 | 262.2 (-1,862.8 ; 2,387.2) | 0.807 | 308.0 (-1,814.7 ; 2,430.7) | 0.775 |  | 206.1 (-1,901.7 ; 2,314.0) | 0.847 | 202.5 (-1,905.7 ; 2,310.7) | 0.85 |
| Quintile 4-5 | 636.7 (-920.6 ; 2,194.0) | 0.42 | 650.5 (-902.4 ; 2,203.4) | 0.409 |  | 541.3 (-992.7 ; 2,075.3) | 0.486 | 559.4 (-974.1 ; 2,093.0) | 0.472 |
| **Model statistics** |  |  |  |  |  |  |  |  |  |
| Number of subjects | 119 |  | 119 |  |  | 118 |  | 119 |  |
| Number of observations | 339 |  | 339 |  |  | 339 |  | 339 |  |
| PSMATCH Infos | K=10, |  | K=11, |  |  | K=15, |  | K=16, |  |

Supplemental table 8 : Sensitivity analysis to assess the impact of associated variables in the multivariate analysis model. Age at start of infertility treatment was successively replaced by year of cancer diagnosis and age at cancer diagnosis.

| **Variable** | **Coef (95%CI)** | **p-val** | **Variable** | **Coef (IC95%)** | **p-val** | **Variable** | **Coef (IC95%)** | **p-val** |
| --- | --- | --- | --- | --- | --- | --- | --- | --- |
| **Time since start of follow up** |  | **<0.001** | **Time since start of follow up** |  | **<0.001** | **Time since start of follow up** |  | **<0.001** |
| Year 1 | 2634.9 [1375.5; 3894.2] | <0.001 | Year 1 | 2642.4 [1412.5; 3872.2] | <0.001 | Year 1 | 2652.5 [1414.0; 3891.1] | <0.001 |
| Year 2 | 2476.6 [1157.1; 3796.2] | <0.001 | Year 2 | 2478.8 [1167.5; 3790.1] | <0.001 | Year 2 | 2487.4 [1164.9; 3809.8] | <0.001 |
| Year 3 | 259.5 [-714.0; 1233.1] | 0.599 | Year 3 | 260.2 [-705.2; 1225.7] | 0.595 | Year 3 | 263.2 [-701.4; 1227.7] | 0.59 |
| Year 4 | Ref |  | Year 4 | Ref |  | Year 4 | Ref |  |
| **Type of cancer treatment** |  | **0.033** | **Type of cancer treatment** |  | **0.027** | **Type of cancer treatment** |  | **0.012** |
| No radiotherapy or chemotherapy | Ref |  | No radiotherapy or chemotherapy | Ref |  | No radiotherapy or chemotherapy | Ref |  |
| Radiotherapy, chemotherapy | 2132.5 [-36.6; 4301.6] | 0.054 | Radiotherapy, chemotherapy | 2295.5 [163.8; 4427.2] | 0.035 | Radiotherapy, chemotherapy | 2209.0 [180.3; 4237.7] | 0.033 |
| Radiotherapy | 4381.6 [1102.0; 7661.3] | 0.009 | Radiotherapy | 4506.3 [1279.2; 7733.4] | 0.007 | Radiotherapy | 4381.1 [1489.6; 7272.6] | 0.003 |
| Chemotherapy | 2017.5 [97.3; 3937.8] | 0.04 | Chemotherapy | 2253.5 [281.1; 4226.0] | 0.025 | Chemotherapy | 2043.9 [288.2; 3799.7] | 0.023 |
| **Quintile FDEP 13** |  | **0.72** | **Quintile FDEP 13** |  | **0.556** | **Quintile FDEP13** |  | **0.299** |
| Quintile 1-2 | Ref |  | Quintile 1-2 | Ref |  | Quintile 1-2 | Ref |  |
| Quintile 3 | 262.2 [-1862.8; 2387.2] | 0.807 | Quintile 3 | 560.9 [-1585.2; 2707.0] | 0.606 | Quintile 3 | 815.6 [-1294.9; 2926.1] | 0.446 |
| Quintile 4-5 | 636.7 [-920.6; 2194.0] | 0.42 | Quintile 4-5 | 835.1 [-700.1; 2370.2] | 0.284 | Quintile 4-5 | 1214.3 [-339.7; 2768.2] | 0.125 |
| **Age at start of infertility treatment** |  | **0.469** | **Year of cancer diagnosis** |  | **0.266** | **Age at cancer diagnosis** |  | **0.295** |
| 18-24 | Ref |  | 1989 and under | Ref |  | 0-1 | Ref |  |
| 25-30 | 391.1 [-2961.9; 3744.1] | 0.818 | 1990 and over | 814.5 [-634.1; 2263.0] | 0.268 | 2-4 | -1451.9 [-3654.4; 750.7] | 0.194 |
| 31-35 | 288.8 [-3185.0; 3762.6] | 0.87 |  |  |  | 5-9 | -615.4 [-3020.6; 1789.7] | 0.614 |
| 36-42 | -1108.2 [-4666.7; 2450.3] | 0.539 |  |  |  | 10-14 | 754.0 [-1682.5; 3190.5] | 0.541 |
|  |  |  |  |  |  | >14 | -189.6 [-2854.7; 2475.4] | 0.888 |
|  |  |  |  |  |  |  |  |  |
| **Model statistics** |  |  | **Model statistics** |  |  | **Model statistics** |  |  |
| Number of subjects | 119 |  | Number of subjects | 119 |  | Number of subjects | 119 |  |
| Number of observations | 339 |  | Number of observations | 339 |  | Number of observations | 339 |  |
| PSMATCH Infos | K=10, Caliper=0.4 |  | PSMATCH Infos | K=10, Caliper=0.4 |  | PSMATCH Infos | K=10, Caliper=0.4 |  |
